# Gender Difference in the Relationships between Inflammatory Markers, Serum Uric Acid and Framingham Risk Score

**DOI:** 10.3390/ijerph18137103

**Published:** 2021-07-02

**Authors:** Jui-Hua Huang, Ren-Hau Li, Shu-Ling Huang, Hon-Ke Sia, Chao-Hung Yu, Feng-Cheng Tang

**Affiliations:** 1 Department of Golden-Ager Industry Management, Chaoyang University of Technology, Taichung 413, Taiwan; Juihua55@ms35.hinet.net; 2 Occupational Health Center, Changhua Christian Hospital, Changhua 500, Taiwan; 3 Department of Psychology, Chung Shan Medical University, Taichung 402, Taiwan; davidrhlee@yahoo.com.tw (R.-H.L.); shuling@csmu.edu.tw (S.-L.H.); 4 Room of Clinical Psychology, Chung Shan Medical University Hospital, Taichung 402, Taiwan; 5 Division of Endocrinology and Metabolism, Changhua Christian Hospital, Changhua 500, Taiwan; 90279@cch.org.tw; 6 Department of Healthcare Administration, Asia University, 500 Lioufong Rd., Wufeng, Taichung 41354, Taiwan; 7 Division of Cardiovascular Medicine, Department of Internal Medicine, Changhua Christian Hospital, Changhua 500, Taiwan; 129887@cch.org.tw; 8 Department of Leisure Services Management, Chaoyang University of Technology, Taichung 413, Taiwan; 9 Department of Occupational Medicine, Changhua Christian Hospital, Changhua 500, Taiwan; 10 School of Medicine, Kaohsiung Medical University, Kaohsiung 807, Taiwan

**Keywords:** Framingham Risk Score, high-sensitivity C-reactive protein, white blood cell, serum uric acid, cardiovascular disease, gender

## Abstract

The purpose of the present study was to explore the role of gender in the relation of high-sensitivity C-reactive protein (hsCRP), white blood cell (WBC) count, and serum uric acid (UA) to the risk of future cardiovascular disease (CVD) events. In total, 404 workers were recruited to obtain the measurements of serum markers for CVD risk. Demographic data, nutrition, exercise, smoking, and alcohol consumption were assessed through a questionnaire. The Framingham Risk Score (FRS) was adopted to estimate the risk of future CVD events. Multiple linear regression models were used to determine CVD risk markers in relation to the FRS by gender. The hsCRP was not significantly correlated with the FRS for all workers after adjusting for covariates, including demographic data and health-related lifestyle. WBC count was positively correlated with FRS for all workers, but WBC count did not show an interaction with gender with respect to the FRS. Serum UA showed an interaction with gender on the FRS, and UA positively correlated with the FRS in males though not in females. With respect to CVD prevention, the WBC count can be used to monitor the risk for all workers. Due to a gender difference shown in the relationship between serum UA and the FRS, serum UA can be a monitor of the risk of future CVD events in male workers only.

## 1. Introduction

Cardiovascular diseases (CVDs) are the leading causes of mortality worldwide. An estimated 31% of all global deaths were from CVDs in 2016 [1]. Among the 10 leading causes of mortality in Taiwan in 2019, malignant tumor ranked first, followed by cardiovascular-related diseases, including heart disease, cerebrovascular disease, diabetes, and hypertension, ranking second, fourth, fifth, and eighth, respectively [2]. These CVD-related diseases also were the top six causes of death in Taiwanese workers [3]. Therefore, accurate prediction of the risk of future CVD events is an important initial step toward preventing CVD-related mortality for worksite health promotion programs in Taiwan. The Framingham Risk Score (FRS) is the most widely used tool to estimate an individual’s 10-year risk of developing cardiovascular events [4,5]. According to the FRS assessment, research has suggested that a 10-year CVD risk can be calculated with approximately 75% accuracy [6]. This provides insight into the noticeable benefits of predicting the risk of future CVD events for clinical or primary prevention [4,7].

Inflammation is considered to be an important mechanism in the pathogenesis of atherosclerosis [8]. Inflammation is also a key factor in the development of CVD-related diseases such as hypertension, stroke, heart failure, vascular dysfunction, and ischemic heart disease [9,10,11]. Accordingly, inflammatory markers have now emerged as significant factors for estimating CVD risk. The acute phase reactant C-reactive protein (CRP), an early inflammatory marker synthesized in the liver, has been widely used in the prediction of people at risk of developing CVDs [10,12,13]. Even though elevated levels of CRP have been found to be associated with CVDs and mortality [10,14,15], their preventive applicability with respect to future cardiovascular events is still unclear.

The use of peripheral blood to detect the role of serum inflammatory markers is increasing because cardiac-imaging methods such as ultrasonography, computed tomography angiography, positron emission tomography, and magnetic resonance images are expensive to use to assess vascular inflammation changes [16]. The white blood cell (WBC) count is an indicator of cellular responses to inflammation [13]. The WBC count, which may be associated with CVD-related morbidity and mortality, has been recognized as a nontraditional risk factor of CVDs [13,17,18,19]. In general, the WBC count may be used as an easy-to-measure, low-cost marker of inflammation, atherosclerosis, and CVD risk [13,17]. The WBC count also has predictive value for the risk of existing cardiovascular events [18,19]. However, it remains unclear whether the WBC count may apply to assessment of the risk of future cardiovascular events according to the FRS.

Recently, growing evidence suggests that elevated serum uric acid (UA) levels may increase the risk of cardiovascular morbidity and mortality [20,21]. Serum UA is associated with inflammation [22], endothelial dysfunction, and hypercoagulability, which may contribute to the development of CVDs [20,23]. The serum UA may play an important role in the pathogenesis of metabolic syndrome and diabetes [23,24]. Additionally, serum UA is related to the development of hypertension as well as to stroke, heart failure, and CVD mortality [25,26]. Although serum UA is associated with CVD-related events and mortality [20,21,23,25,26], only a minority of studies have explored the relationship of serum UA and future CVD events according to the FRS [27,28].

Gender differences in CVD pathophysiology, clinical presentation, prevention, and management have been observed [29,30,31]. Lifestyle may play an important role in the gender difference in the development of CVDs [31]. Males usually have poorer nutritional habits than females [32]. Females usually have a lower rate of alcohol consumption and smoking compared to males [33,34]. Sex hormones also may affect the development of CVDs in males and females [31]. In addition, animal tests found gender differences in the functions of vascular endothelial cells and oxidant/antioxidant responses under oxidative stress conditions [35,36]. However, very few studies have investigated whether the relationships between inflammation markers, serum UA, and the risk of future CVD events may have gender-specific effects. The purpose of this study was to explore the role of gender in the relation of high-sensitivity CRP (hsCRP), WBC count, and serum UA to the risk of future CVD events.

## 2. Materials and Methods

### 2.1. Ethics Statement

Ethical approval for this study was obtained from the Institutional Review Board of the Changhua Christian Hospital in Taiwan (CCHIRB No: 120606 and 191238). The study was conducted according to the principles of the Declaration of Helsinki. The candidates were free to decide whether to join in the study. The voluntary participants completed the questionnaire and clinical variable measurements as requested for the study. The questionnaires were returned along with the participants’ clinical variables under encoding by each contacted company’s occupational health personnel. Accordingly, all data collected during the study remained anonymous and were kept strictly confidential to protect the privacy of the participants.

### 2.2. Study Sample

This cross-sectional study was conducted in 2012–2013 by the Center for Occupational Health. The research design used a convenience sampling method to select the participants. Workers aged 20 years or over were recruited from two manufacturers of pumps and automotive parts in central Taiwan to take part in the study. The selection of manufacturers, based on proven positive relationships with the Center for Occupational Health, allowed the study to proceed without problems. A total of 648 individuals were candidates for the study; a multiple imputation method for missing values was executed, and complete data including 446 male and 202 female workers were obtained. In order to balance the sample size and ensure similar health status for the two gender groups, propensity score matching was adopted based on certain blood biochemical data, including serum glutamate pyruvate transaminase (SGPT), serum glutamic oxaloacetic transaminase (SGOT), fasting blood glucose (FBG), low-density lipoprotein (LDL), and blood urea nitrogen (BUN). Considering the statistical rationality, the covariates used in the following multivariable method were not used as matched variables in the implementation of the matching. In total, 404 participants including 202 male and 202 female were then selected out.

### 2.3. Demographic Data and Health-Related Lifestyle

Demographic data and health-related lifestyle, including smoking, alcohol consumption, exercise, and nutritional health behaviors, were obtained through a self-reported questionnaire. Each participant’s smoking status was classified as without smoking and with smoking (including occasional or daily smoking). Alcohol consumption was classified as without alcohol drinking and with alcohol drinking (including occasional or daily alcohol drinking). In addition, the information on exercise and nutritional health behaviors was obtained using the subscales of Health-Promoting Lifestyle Profile II (Taiwanese version). Participants were asked to rate nine items for nutritional health behavior and eight items for exercise health behavior on a four-point Likert scale (Never, Sometimes, Often, and Routinely). A higher score indicated a greater level of participation in exercise and nutritional health behaviors. In addition, clinical variables, including anthropometric measurements and markers for CVD risk, were collected through the identified manufacturers’ annual health screening required by Taiwanese regulations.

### 2.4. Anthropometric and Blood Pressure Measurements

The body mass index (BMI) was calculated according to weight in kilograms divided by height in meters squared (kg/m^2^). The blood pressure (BP) measurement was taken using a validated digital sphygmomanometer (HEM-7310, Omron, Kyoto, Japan) while the participant was in a seated position after at least five minutes of rest. The procedure was performed in accordance with the practice guidelines of the European Society of Hypertension for blood pressure measurement [37].

### 2.5. Measurements of Blood Lipids, UA, and Inflammatory Markers

For biochemical determinations of blood, participants were asked to fast for 8–10 h the night prior to measurement. Study measurements were performed by the hospital medical laboratory (certified ISO15189) using standard methods. Serum lipids and UA were tested using a Dimension RxL autoanalyzer (Siemens, Newark, NJ, USA). Total cholesterol and high-density lipoprotein cholesterol (HDL-C) were assessed using standard enzymatic methods. Serum UA levels were assessed using the uricase method. Additionally, the hsCRP and WBC count were measured as inflammatory markers. The hsCRP was measured by particle-enhanced turbidimetric immunoassay. The WBC count was measured by the direct current detection method (XT1800i, Sysmex, Kobe-shi, Hyogo, Japan).

### 2.6. Calculation of Framingham Risk Score

The risk of future CVD events was assessed by the FRS. This study calculated the FRS according to the criteria of the third report of the National Cholesterol Education Program Adult Treatment Panel (NCEP-ATP III) [38]. Calculation of the total risk score took into consideration the risk factors of sex, age, smoking, total cholesterol, HDL-C, systolic BP, and whether any anti-hypertensive treatments were prescribed. Then, the scores for each factor were estimated according to the Framingham risk table for both genders, and the overall risk scores were then calculated by summing the scores for each risk factor.

### 2.7. Statistical Analysis

The 2-tailed t-test was used for continuous data with mean ± standard deviation. The Chi-squared test was used for the relationships in categorical data with number and percentage. The Pearson correlation coefficient was used for the degree of linear relationship between two continuous variables. The Phi correlation was used for the correlation between two binary variables. The point-biserial correlation was used for the correlation between a binary variable and a continuous variable. The relationship between the FRS and CVD risk markers was examined in the simple and multiple regression analysis. The CVD risk markers, including hsCRP, WBC count, and serum UA, were centered from their means to form their interaction terms with gender in the multiple regression analysis. When an interaction term showed significance, further multiple regression analysis of the FRS for each gender was conducted separately. All outcomes of the regression analysis are presented in unstandardized coefficient (B) and standardized coefficient (β), at a 95% Confidence Interval for B. All statistical procedures were performed using SPSS (version 23.0) statistical software (SPSS Inc., Chicago, IL, USA), and a *p*-value less than 0.05 was considered statistically significant.

## 3. Results

### 3.1. Descriptive Statistics of the Participants by Gender

Table 1 shows the participants’ characteristics. Of the 404 participants, 202 (50.0%) were male and 202 (50.0%) were female. Age, smoking status, alcohol consumption, nutritional behavior, HDL-C, Systolic BP, FRS, and serum UA showed a gender-based difference. Male workers had higher levels on the FRS and of serum UA compared to the females (*p* < 0.001). Moreover, male workers had a significantly higher percentage of smoking and alcohol drinking and a lower nutritional behavior score than the females (*p* < 0.001). Overall, the results showed male workers had higher risk of CVD compared to the females. Moreover, male workers had less healthy lifestyles than female workers.

### 3.2. Crude Correlations between FRS, Age, BMI, Health-Related Lifestyle, and CVD Risk Markers

The crude correlations between FRS, age, BMI, health-related lifestyle, and CVD risk markers for male and female workers are summarized in Table 2 and Table 3, respectively. Table 2 shows a positive correlation between FRS and age (r = 0.603, *p* < 0.001), BMI (r = 0.173, *p* < 0.05), smoking (r = 0.295, *p* < 0.001), nutrition (r = 0.149, *p* < 0.05), exercise (r = 0.187, *p* < 0.01), and WBC count (r = 0.199, *p* < 0.01) for the males. However, FRS was not significantly correlated with alcohol consumption, hsCRP, and serum UA. In Table 3, for the females, there was a positive correlation between FRS and age (r = 0.642, *p* < 0.001) and serum UA (r = 0.179, *p* < 0.05). However, FRS was not significantly correlated with the BMI, hsCRP, WBC count, and health-related lifestyle, including smoking status, alcohol consumption, nutrition, and exercise. In general, the correlations between FRS and CVD risk markers showed a gender difference. In addition, the crude correlations between hsCRP and WBC count were statistically significant for both genders.

### 3.3. Simple and Multiple Linear Regression Models for FRS in Relation to CVD Risk Markers

The results of simple and multiple linear regression analysis for the relationships between FRS and hsCRP, WBC count, and serum UA are summarized in Table 4. The hsCRP did not show a statistical significance on the FRS for all workers. The WBC count was observed to have a significant positive correlation with FRS for all workers (B = 0.185, β = 0.104, *p* = 0.043), but the WBC count did not show any significant interaction with gender on the FRS. Serum UA did not show statistical significance on the FRS for all workers. Nevertheless, the interaction between gender and serum UA on the FRS showed a statistical significance (B = 0.551, β = 0.172, *p* = 0.002). In further analysis, serum UA was significantly positively correlated with FRS for the male workers (B = 0.583, β = 0.186, *p* < 0.001). However, serum UA was not significantly associated with FRS for the female workers.

## 4. Discussion

This study aimed to investigate the relation of the risk of future CVD to hsCRP, WBC count, and serum UA, especially with respect to the role of gender. Our data suggested that, using multiple linear regression analysis, (1) clear gender difference was observed in the relationship between serum UA and FRS. Serum UA positively correlated with FRS in male workers, but not in female workers; (2) WBC count positively correlated with FRS for all workers; and (3) hsCRP did not show a significant association with FRS for both genders.

Growing evidence suggests that elevated serum UA levels may increase the risk of cardiovascular morbidity and mortality [20,21]. The present study showed that serum UA levels are positively associated with FRS in males after adjusting for covariates. A longitudinal aging study in Taiwan showed that raised serum UA levels were associated with high FRS after adjustment for potential risk factors [39]. A previous study indicated that the 10-year CVD risk gradually augmented with an increase in the levels of circulating UA [40]. Data from another study also revealed that an increase in serum UA levels was associated with an increase in CVD risk by the FRS [28]. These previous studies suggest that elevated serum UA levels were associated with the risk of future CVD events. The reason for serum UA being related to FRS may be that UA is associated with endothelial dysfunction, inflammation, and hypercoagulability [20,22,23]. Studies have shown that UA molecules reduce the bioavailability of nitric oxide in vascular endothelial cells and increase reactive oxygen species. The generated oxidative pressure causes damage to the inner wall of blood vessels, which may lead to changes in the blood flow of vessels. UA can also cause autoinflammation, innate immunity, and activation of the renin-angiotensin-aldosterone system. These pathogenic mechanisms may contribute to the development of future cardiovascular events [20,22,23]. Moreover, the present study found that serum UA had significant interaction with gender for predicting FRS. Serum UA and FRS showed a positive association in males but not in females. Possible explanations for this finding are that sex hormones may play important roles in this association. The majority of the participants in the present study were younger adults. Normally, compared with males, females have a higher plasma estrogen level that may increase the renal clearance of serum UA and have a protective effect of reducing cardiovascular risk [41,42]. In addition, animal experiments in rats found that UA is related to oxidative stress, under which female rats can release more nitric oxide and antioxidants to regulate the function of the vascular endothelium [36]. Moreover, the vascular endothelial cells of male rats have poorer relaxation ability [35]. This may contribute to the association between serum UA and the risk of future CVD events found in the males but not in the females. The present study suggested that serum UA not only is an important monitor but also shows a gender difference in the relation to the risk of future CVD events. This finding remains a matter for further exploration.

Past research on the association between the WBC count and cardiovascular events found that the WBC count is an independent risk factor for all-cause mortality and for all atherosclerotic CVD mortality [18]. In addition, a higher WBC count was related to carotid and femoral atherosclerosis [17]. The present study, using multiple linear regression analysis, showed that the WBC count was significantly positively correlated with FRS for all workers. Similar findings from several studies also showed the WBC count was positively associated with a relatively high 10-year CVD risk according to FRS [43,44]. The present study extends the previous findings to further support the association between a high WBC count and the risk of future CVD events. The WBC count is a major mediator of inflammation and plays a central *role* in host defense and tissue injury [45]. The WBC may act through several mechanisms, including the abnormal WBC aggregation, activation of the coagulation system (which is related to atherosclerotic risk factor), formation of the platelet-leukocyte aggregation, microvascular plugging, and microvasculature obstruction. These inflammatory reactions may all contribute to cardiovascular events [45]. In clinical practice, the WBC count has been recognized as a nontraditional risk factor for CVDs and has predictive value for cardiovascular events [13,17,18]. The present study supported the idea that the WBC count could be an easy-to-measure, low-cost marker of the risk of future CVD events with respect to worksite health promotion strategies. Moreover, using the WBC count to estimate the risk of future CVD events can be applied to both genders.

The CRP in relation to cardiovascular events and mortality has been supported in several studies [10,13,14,15]. Regarding the preventive applicability of CRP in the prediction of the risk of future CVD events, a previous observational study indicated that elevated CRP was associated with increased 10-year CVD risk using the FRS in older men and women [46]. A patient-based prospective study also showed that an increased 10-year CVD risk using FRS was associated with an elevated level of CRP [47]. In contrast, the present study revealed that the hsCRP was not associated with 10-year CVD risk using the FRS when serum UA and WBC count were taken into consideration simultaneously. A possible reason for this finding is that the prediction of hsCRP for the risk of future CVD events was reduced or suppressed by the WBC count. In the present study, there were positive correlations between the WBC count and hsCRP for both genders (Table 2 and Table 3). The WBC count and hsCRP are important factors in inflammatory response [48]. When the innate immune system is activated, macrophages (a type of WBC) secrete pro-inflammatory cytokines and are involved in the up-regulation of the inflammatory response [47,48,49,50]. An elevated level of pro-inflammatory cytokines stimulates the liver to synthesize and secrete CRP [49,50,51,52]. It is conceivable that the prediction of the risk of future CVD events based on hsCRP is likely to be affected by the WBC count. The present study suggested that the WBC count should be considered when exploring whether the prediction of the risk of future CVD events based on hsCRP has preventive applicability.

This study reveals some important findings for identifying employees at high risk for CVDs. For policy implications of worksite health promotion, it is suggested that serum UA could be a useful marker of the risk of future CVD events with respect to male workers. The WBC count can be used to monitor the risk of future CVD events for all workers. Although this study shed light on the relationships between the risk of future CVD events and inflammatory markers and serum UA by gender, this study had two main limitations. First, this was a cross-sectional study. Though we found that the relation of the risk of future CVD events to hsCRP, WBC count, and serum UA showed different significance by gender, we could not establish the exact causal relationship between predictors and CVDs. The prediction of future CVD events should be validated by follow-up research. More longitudinal studies are recommended. Second, menstrual status and estrogen replacement were not included in the personal information. These factors may confound the relationship between predictors and CVD risk, especially in female workers. Therefore, further research that includes information on sex hormones is recommended. 

## 5. Conclusions

Identifying high-risk individuals is important for CVD prevention at the workplace. In relation to the risk of future CVD events, serum UA and inflammatory markers showed different significance by gender. Therefore, it is suggested that serum UA could be an available marker of the risk of future CVD events in male workers. The WBC count can be used to monitor the risk of future CVD events for all workers. Additionally, hsCRP may be an invalid indicator to estimate the risk of future CVD events when simultaneously considering the WBC count and serum UA.

## Figures and Tables

**Table 1:** Descriptive statistics of the participants by gender.

Variables ^1^	Total *n* = 404	Male *n* = 202	Female *n* = 202	*p* ^2^
Age (y)	39.5 ± 9.0	37.6 ± 9.2	41.4 ± 8.3	<0.001
BMI (kg/m^2^)	23.6 ± 3.6	23.4 ± 3.4	23.8 ± 3.8	0.363
Smoking				
With	71 (17.6)	66 (32.7)	5 (2.5)	<0.001
Without	333 (82.4)	136 (67.3)	197 (97.5)	
Alcohol				
With	118 (29.2)	90 (44.6)	28 (13.9)	<0.001
Without	286 (70.8)	112 (55.4)	174 (86.1)	
Nutrition behavior score	2.4 ± 0.4	2.3 ± 0.4	2.5 ± 0.4	<0.001
Exercise behavior score	1.7 ± 0.5	1.7 ± 0.5	1.7 ± 0.4	0.725
Total cholesterol (mg/dL)	193.6 ± 36.1	190.2 ± 34.7	197.1 ± 37.3	0.055
HDL-C (mg/dL)	57.5 ± 16.6	53.9 ± 11.7	61.0 ± 13.4	<0.001
Systolic BP (mmHg)	123.3 ± 16.6	126.5 ± 14.9	120.2 ± 17.6	<0.001
FRS (%)	1.7 ± 3.2	3.0 ± 4.0	0.3 ± 0.7	<0.001
hsCRP (mg/L)	1.3 ± 2.2	1.4 ± 2.7	1.3 ± 1.6	0.461
WBC (10^3^ cells/µL)	6.8 ± 1.8	6.9 ± 1.7	6.7 ± 1.9	0.207
Serum UA (mg/dL)	5.8 ± 1.5	6.6 ± 1.7	5.1 ± 1.3	<0.001

^1^ Abbreviations: BMI, body mass index; HDL-C, high-density lipoprotein cholesterol; BP, blood pressure; FRS, Framingham risk score; hsCRP, high-sensitivity C-reaction protein; WBC, white blood cell; UA, uric acid. ^2^ The 2-tailed t-test was used for continuous data with mean ± standard deviation. Chi-squared test was used for the relationships in categorical data with number and percentage, *n* (%). Significance level is *p* < 0.05.

**Table 2:** Crude correlations between FRS, age, BMI, health-related lifestyle, and CVD risk markers in male workers.

Variables ^1^	FRS	^2^	Age	^2^	BMI	^2^	Smoking	^2^	Alcohol	^2^	Nutrition	^2^	Exercise	^2^	hsCRP	^2^	WBC	^2^
FRS (%)	--																	
Age (years)	**0.603**	***	--			*												
BMI (kg/m^2^)	**0.173**	*	**0.161**		--													
Smoking	**0.295**	***	**−0.177**	*	−0.081		--											
Alcohol	0.123		−0.035		0.112		**0.247**	**	--									
Nutrition	**0.149**	*	**0.236**	**	0.129		−0.044		0.083		--							
Exercise	**0.187**	**	**0.259**	***	−0.004		−0.047		0.047		**0.349**	***	--					
hsCRP (mg/L)	0.134		−0.012		**0.337**	***	0.128		0.015		0.083		−0.041		--			
WBC (10^3^ cells/µL)	**0.199**	**	−0.001		**0.236**	**	**0.242**	**	0.118		−0.096		−0.050		**0.319**	***	--	
Serum UA (mg/dL)	0.034		**0.240**	**	**0.294**	***	0.016		**0.157**	*	0.016		0.027		0.040		0.114	

^1^ Abbreviations: FRS, Framingham risk score; BMI, body mass index; hsCRP, high-sensitivity C-reaction protein; WBC, white blood cell; UA, uric acid. ^2^ The correlation coefficients were calculated by Pearson correlation, Phi correlation, or Point-biserial correlation. Pearson correlation was used for the correlation between continuous variables. Phi correlation was used for the correlation between two binary variables. Point-biserial correlation was used for the correlation between a binary variable and a continuous variable. Significant values shown in bold in heatmap. * *p* < 0.05; ** *p* < 0.01; *** *p* < 0.001.

**Table 3:** Crude correlations between FRS, age, BMI, health-related lifestyle, and CVD risk markers in female workers.

Variables ^1^	FRS	^2^	Age	^2^	BMI	^2^	Smoking	^2^	Alcohol	^2^	Nutrition	^2^	Exercise	^2^	hsCRP	^2^	WBC	^2^
FRS (%)	--																	
Age (years)	**0.642**	***	--															
BMI (kg/m^2^)	0.081		−0.017		--													
Smoking	0.014		**−0.226**	**	**0.181**	*	--											
Alcohol	−0.012		−0.039		**0.172**	*	0.021		--									
Nutrition	−0.041		−0.002		−0.083		**−0.084**	*	0.092		--							
Exercise	0.128		0.117		0-.055		−0.007		−0.076		**0.183**	**	--					
hsCRP (mg/L)	0.115		0-.015		**0.478**	***	**0.145**	*	0.049		−0.109		−0.059		--			
WBC (10^3^ cells/µL)	−0.037		**−0.213**	**	**0.384**	***	−0.007		−0.019		0.057		−0.023		**0.344**	***	--	
Serum UA (mg/dL)	**0.179**	*	**0.165**	*	**0.318**	***	0.032		0.129		−0.005		0.084		0.119		0.114	

^1^ Abbreviations: FRS, Framingham risk score; BMI, body mass index; hsCRP, high-sensitivity C-reaction protein; WBC, white blood cell; UA, uric acid. ^2^ The correlation coefficients were calculated by Pearson correlation, Phi correlation, or Point-biserial correlation. Pearson correlation was used for the correlation between continuous variables. Phi correlation was used for the correlation between two binary variables. Point-biserial correlation was used for the correlation between a binary variable and a continuous variable. Significant values shown in bold in heatmap. * *p* < 0.05; ** *p* < 0.01; *** *p* < 0.001.

**Table 4:** Simple and multiple linear regressions for FRS in relation to CVD risk markers.

	FRS (%) ^2,4^ (Separate Analysis)				FRS (%) ^3,4^ (Simultaneous Analysis)			
Variables ^1^	B	β	*p*	95.0% CI for B	B	β	*p*	95.0% CI for B
(Constant)					−9.479		<0.001	(−11.762, −7.196)
Gender	**2.653**	**0.419**	<0.001	(2.089, 3.218)	**2.316**	**0.366**	<0.001	(1.755, 2.878)
Age (years)	**0.129**	**0.365**	<0.001	(0.097, 0.161)	**0.196**	**0.554**	<0.001	(0.169, 0.222)
BMI (kg/m^2^)	0.081	0.093	0.062	(−0.004, 0.166)	0.025	0.029	0.485	(−0.046, 0.096)
Smoking	**3.307**	**0.397**	<0.001	(2.558, 4.056)	**2.905**	**0.349**	<0.001	(2.248, 3.562)
Alcohol	**1.508**	**0.229**	<0.001	(0.878, 2.137)	0.144	0.022	0.569	(−0.353, 0.641)
Nutrition behavior score	−0.034	−0.005	0.924	(−0.734, 0.666)	0.156	0.022	0.559	(−0.369, 0.682)
Exercise behavior score	**0.860**	**0.134**	0.007	(0.234, 1.485)	0.294	0.046	0.210	(−0.167, 0.755)
hsCRP (mg/L)	**0.184**	**0.127**	0.011	(0.043, 0.324)	−0.057	−0.039	0.604	(−0.272, 0.158)
WBC (10^3^ cells/µL)	**0.250**	**0.141**	0.004	(0.078, 0.422)	**0.185**	**0.104**	0.043	(0.006, 0.364)
Serum UA (mg/dL)	**0.538**	**0.251**	<0.001	(0.335, 0.742)	−0.194	−0.091	0.131	(−0.446, 0.058)
Gender × hsCRP ^5^					0.143	0.085	0.244	(−0.098, 0.385)
Gender × WBC ^5^					0.013	0.005	0.923	(−0.254, 0.280)
Gender × Serum UA ^5^					**0.551**	**0.172**	0.002	(0.201, 0.901)
Male								
(Constant)					−11.391		<0.001	(−14.678, −8.103)
Age (years)					**0.313**	**0.715**	<0.001	(0.267, 0.359)
BMI (kg/m^2^)					0.044	0.037	0.470	(−0.075, 0.163)
Smoking					**3.586**	**0.419**	<0.001	(2.751, 4.420)
Alcohol					0.104	0.014	0.780	(−0.626, 0.833)
Nutrition behavior score					−0.162	−0.018	0.730	(−1.087, 0.762)
Exercise behavior score					0.164	0.022	0.673	(−0.601, 0.928)
Serum UA (mg/dL)					**0.583**	**0.186**	<0.001	(0.259, 0.908)
Female								
(Constant)					−2.034		<0.001	(−2.819, −1.249)
Age (years)					**0.052**	**0.662**	<0.001	(0.043, 0.060)
BMI (kg/m^2^)					0.009	0.053	0.366	(−0.011, 0.028)
Smoking					**0.639**	**0.153**	0.007	(0.175, 1.102)
Alcohol					−0.024	−0.013	0.817	(−0.229, 0.181)
Nutrition behavior score					−0.047	−0.031	0.579	(−0.213, 0.119)
Exercise behavior score					0.081	0.055	0.317	(−0.078, 0.240)
Serum UA (mg/dL)					0.024	0.045	0.434	(−0.036, 0.083)

^1^ Abbreviations: FRS, Framingham risk score; BMI, body mass index; hsCRP, high-sensitivity C-reaction protein; WBC, white blood cell; UA, uric acid. ^2^ Simple linear regression analysis. ^3^ Multiple linear regression analysis. ^4^ All outcomes of the multiple regression analysis are presented in unstandardized coefficient (B) and standardized coefficient (β), at a 95% Confidence Interval (CI) for B. Unstandardized coefficient (B) represents the effect of one-unit change in the explanatory variable for FRS. For example, in the male group, as the serum UA increases one unit, FRS increases by 0.398%. ^5^ Explanatory variables including hsCRP, WBC count, and Serum UA were centered from their means to form their interaction terms with gender in the multiple regression analysis. Significant values shown in bold in heatmap.

## Data Availability

The data are not publicly available due to privacy and ethical restrictions.
